# Elastic MCF Rubber with Photovoltaics and Sensing on Hybrid Skin (H-Skin) for Artificial Skin by Utilizing Natural Rubber: 2nd Report on the Effect of Tension and Compression on the Hybrid Photo- and Piezo-Electricity Properties in Wet-Type Solar Cell Rubber

**DOI:** 10.3390/s18061848

**Published:** 2018-06-06

**Authors:** Kunio Shimada

**Affiliations:** Faculty of Symbiotic Systems Sciences, Fukushima University, 1 Kanayagawa, Fukushima 960-1296, Japan; shimadakun@sss.fukushima-u.ac.jp; Tel.: +81-24-548-5214

**Keywords:** hybrid skin (H-Skin), solar cell, sensing, piezoelectricity, photovoltaics, natural rubber, electrolytic polymerization, magnetic cluster, magnetic field, magnetic compound fluid (MCF), artificial skin, robot, humanoid

## Abstract

In contrast to ordinary solid-state solar cells, a flexible, elastic, extensible and light-weight solar cell has the potential to be extremely useful in many new engineering applications, such as in the field of robotics. Therefore, we propose a new type of artificial skin for humanoid robots with hybrid functions, which we have termed hybrid skin (H-Skin). To realize the fabrication of such a solar cell, we have continued to utilize the principles of ordinary solid-state wet-type or dye-sensitized solar rubber as a follow-up study to the first report. In the first report, we dealt with both photovoltaic- and piezo-effects for dry-type magnetic compound fluid (MCF) rubber solar cells, which were generated because the polyisoprene, oleic acid of the magnetic fluid (MF), and water served as p- and n- semiconductors. In the present report, we deal with wet-type MCF rubber solar cells by using sensitized dyes and electrolytes. Photoreactions generated through the synthesis of these components were investigated by an experiment using irradiation with visible and ultraviolet light. In addition, magnetic clusters were formed by the aggregation of Fe_3_O_4_ in the MF and the metal particles created the hetero-junction structure of the semiconductors. In the MCF rubber solar cell, both photo- and piezo-electricity were generated using a physical model. The effects of tension and compression on their electrical properties were evaluated. Finally, we experimentally demonstrated the effect of the distance between the electrodes of the solar cell on photoelectricity and built-in electricity.

## 1. Introduction

Ordinary solar cells are fabricated as a solid-state material and are typically used in the configuration state as a flat plate so that they are difficult to bend, compress by pressure, or expand by tension, and cannot withstand impact and load forces. However, if a solar cell could be made flexible, elastic and extensible, many engineering application concepts could benefit from this development to achieve marked progress. In robotics, for example, it could be used in human-like robots as an artificial skin. No power supply would be required to drive their motors and sensors, because electric power would be generated by the solar cells. In addition, the robot’s skin would be able to move with a supple motion similar to that of humans. We can propose that rubber is one of elasticized materials usable for realizing a flexible solar cell. Flexible solar power generators have been investigated recently [1,2,3,4,5,6,7], although they have remained within the realm of the solid state. This is due to the conducting polymer. Therefore, the effect of tension and compression on photovoltaics has not been investigated. Unfortunately, there have been only a few attempts at using rubber in this field. Investigations utilizing polysiloxane rubber [8] and styrene-butadiene-rubber [9] are the only noteworthy examples. However, these investigations did not clarify the effect of the properties of the rubber, including tension and compression, on the photovoltaics. Induced output, voltage and current were clarified, but the effects of tension and compression have not been elucidated. In the present study, we attempt the fabrication of a solar cell utilizing rubber and to investigate the effect of tension and compression on the photovoltaics.

There are some useful principles to be gleaned from the fabrication process of ordinary solid-state solar cells for the present effort of constructing a flexible, elastic and extensible solar cell. They are primarily categorized in fields concerning the containing components: organic or inorganic material [6,10,11]; organic or inorganic material involving lipids [12]: ionic liquid [1,13,14,15,16,17]: dielectric liquid [18], etc. Among them, the principles of the electrolyte system as a situated wet-type solar cell [19,20] or dye-sensitized one [21,22] such as a Gratzel-type solar cell [23], are particularly useful for the solar cell developed here, as the rubber described below is a water-soluble flux. In addition, low-cost and lightweight conditions are required in the engineering production of solar cells.

In the first report [24], we investigated the possibility of a novel rubber-type solar cell with a sensing, magnetic compound fluid (MCF) rubber solar cell, which correspond to a new type of artificial skin named “hybrid skin (H-Skin)” with the hybrid functions of elasticity, flexibility, photovoltaics and piezoelectricity obtained by synthesis using natural rubber (NR)-latex, MCF, TiO_2_ particles, dye and electrolyte, with reference to the principles of the fabrication of wet-type or dye-sensitized solar cells using a physical-chemical model. MCF is an intelligent fluid that is responsive to a magnetic field, first reported in 2001. It is a colloidal fluid involving 10-nm Fe_3_O_4_ particles coated with oleic acid because of compounding magnetic fluid (MF), and other metal particles such as Fe, Ni or Cu on the order of 1 μm [25]. When NR-latex is included in the MCF (called MCF rubber), it can be solidified under the application of a magnetic field, and magnetic clusters combined by the aggregation of Fe_3_O_4_ and metal particles are formed along the direction of the magnetic field lines. We used a new method for solidification of water-soluble rubber, natural rubber (NR-latex) by electrolytic polymerization with a magnetic field; the electrolytic polymerization is a kind of electrolysis, and NR-latex is water-soluble and has C=C bonds. Therefore, NR-latex can be electrolytically polymerized in a very short time interval. The alignment of the magnetic clusters enhances the photovoltaic properties as well as electric and thermal conductivity. In addition, the polyisoprene of the NR-latex, oleic acid, and water molecules play the roles of p- and n-type semiconductors, as indicated in the first report. Therefore, the MCF rubber solar cell has been shown to have piezoelectric properties based on the built-in voltage of the semiconductor [26], and photovoltaic properties based on the semiconductor. The former is comparable to piezoelectric-like behavior and the latter to solar cell-like one. In addition, it is elucidated that the combination of NR-latex as a water-soluble rubber and TiO_2_ particles could have a photovoltaic effect since the combination of water and TiO_2_ has been reported to have a photovoltaic effect with electrochemical photolysis (called the Honda-Fujishima effect) [27]. Therefore, the composite synthesized by NR-latex, MF and TiO_2_ particles produces both photovoltaics and piezoelectricity. In the first report, the principle of generated photovoltaics of a dry-type MCF solar cell rubber, which is both a photovoltaic and piezoelectric material, was described using a chemical-physical model. On the basis of this principle, the experimental results obtained in the first report are discussed together with the physical and chemical interpretation.

Whether the surface condition of the MCF solar cell rubber is damp or not is a significant factor. “Dry type” refers to dry-produced solar cells: the MCF rubber is vulcanized so that this results in dry rubber solar cells because heat is generated by electrolytic polymerization [28]; the dye and electrolyte are not deposited on the MCF rubber so that they are involved in the MCF rubber liquid before vulcanization. The first report dealt with the dry-type solar cell. In contrast, the resulting solar cell is denoted as “wet type” such as the Gratzel-type solar cell. Naturally, the addition of dye and electrolyte as a wet-type solar cell may be expected to enhance the photovoltaic effect because the dye seeps into the electrolytically polymerized MCF rubber and photovoltaic power is generated by a redox reaction. At the onset of the present report, we deal with wet-type MCF rubber solar cells and discuss the photovoltaic effect by a comparison between dry- and wet-types. 

In the present report, we investigate the effect of several factors on the MCF rubber solar cell under conditions without tension and compression. In the first report, the dry-type MCF rubber solar cell induced a relatively small electric current density. This result was influenced by other experimental conditions such as the kind of sensitized dye and the kind of electrode material caused in work function. Therefore, we argue that these factors of experimental conditions are significant without tensing and compressing a wet-type MCF rubber solar cell. 

Next, the effect of simultaneously existing tension and compression on the photovoltaics of wet-type MCF rubber solar cell is investigated in this report. In the first report, we dealt with the experimental condition of only compression within a small pressure range, and the case of tension effect on the MCF rubber solar cell was not evaluated. The present report deals with large compression as well as tension. On the other hand, in the field of robotics, the condition of simultaneously existing tension and compression is sufficiently significant to warrant the further development of MCF rubber solar cells, because the artificial skin installed in a robot would be elongated and compressed simultaneously in most instances. We then explore the effect of simultaneous excitation of tension and compression on the hybrid properties of photoelectricity (photovoltage and photocurrent) and piezoelectricity in the MCF rubber solar cell.

Finally, we investigate the effect of electrodes location on the changes in voltage and electric current by illumination. For the effective feasibility of MCF rubber solar cells to engineering applications such as robotics, the electrode location becomes a major issue in the design and installation of the MCF rubber solar cell. The present report investigates the effect of electrode location on photovoltaics of the wet-type MCF rubber solar cell without tension and compression.

## 2. Wet-Type MCF Rubber Solar Cell

**Figure 1:**
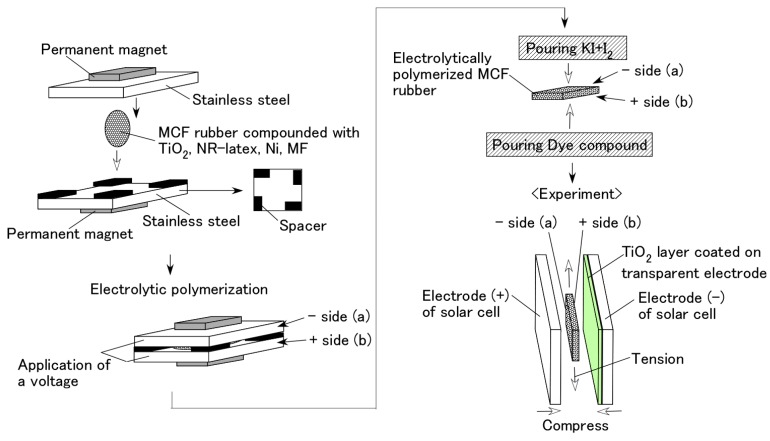
Fabrication procedure of wet-type MCF rubber solar cell.

As shown in Figure 1, the first step in the fabrication of an MCF rubber solar cell involved combining the n-type semiconductor TiO_2_ (titanium with 10–50 nm particle size, anatase form, Fujifilm Wako Pure Chemical Co., Ltd., Osaka, Japan) and MCF rubber, which consist of carbonyl Ni powder with particles on the order of µm and pimples on the surface (No. 123, Yamaishi Co., Ltd., Noda, Japan), water-based MF with 40-wt % Fe_3_O_4_ (W-40 with 10 nm sphere particles, Ichinen-Chemicals Co., Ltd., Shibaura, Japan), and NR-latex (Rejitex Co., Ltd., Atsugi, Japan). An electric field held constant at 6 V and 2.7 A was applied between stainless steel plates with 1-mm gap for 10 min under atmospheric conditions in the region of neodymium permanent magnets with a 188-mT magnetic field strength at the position of the MCF rubber liquid between the electrodes. The eletrolytically polymerized MCF rubber produced was 20 mm × 23 mm ×1 mm. The procedure is the same as the one conducted on by the previous study [28]. After electrolytic polymerization, 0.17-g solution KI + I_2_ (which is mixed by potassium iodide (KI, Fujifilm Wako Pure Chemical Co., Ltd., Osaka, Japan) and iodine (I_2_, Fujifilm Wako Pure Chemical Co., Ltd.) which contained 3.3-g iodine I_2_ in a solution of 40-g potassium iodide KI and 60-g water was used as an electrolyte. In this case, the electrolyte was deposited on one side of the electrolytically polymerized MCF rubber at the cathode (a) in Figure 1, and the liquid dye based on Ruthenium complexes PEC-TOM-P04 (Peccell Technologies Co., Ltd., Yokohama, Japan), was used on the other side of the sample at the anode (b). The surface on side (b) was in contact with a transparent glass coated with TiO_2_, and was connected to a cathode electrode as a solar cell; the surface on (a) was also in contact with a transparent glass to serve as the anode electrode of the solar cell.

Visible light (238 Lux) and ultraviolet light (227 Lux) were scattered on the transparent electrode coated with TiO_2_. The voltage and electric current between the electrodes were measured using a digital multi-meter (PC710, Sanwa Supply Co., Ltd., Okayama, Japan).

MCF rubber solar cell is a synthesis of dye-sensitized solar cell and organic thin-film solar cell, as well as tandem-type solar cell at electrolytic polymerization. The photoelectric reaction of the MCF rubber solar cell was shown in the first report. It can be summarized as follows: during electrolytic polymerization, polyisoprene and water molecules are ionized. As a result, the ionized polyisoprene plays the role of the acceptor such as in the case of p-type semiconductors, corresponding to A^−^ (A is the acceptor). The hydrogen ion decomposed from the water molecule in the NR-latex and MF assume the role of donors similar to the case of n-type semiconductors, corresponding to D^+^ (D is the donor). In the case of ordinary solid-state solar cells, p- and n-type semiconductors are prepared to be ionized in advance. However, in the present MCF rubber solar cell, p- and n-type semiconductors are generated by electrolytic polymerization. Finally, P consisting of polyisoprene of NR-latex and oleic acid of MF is ionized as either positively or negatively and reacts with A^−^ or D^+^. The electron and hole emitted from A and D are then neutralized, and built-in electricity (built-in voltage and built-in current) is generated between semiconductors A^−^ and D^+^. Therefore, the piezo-electric behavior occurs. The amplitude changes with changes due to pressure and tension. The former, in particular, corresponds to piezoelectricity. This photo-mechanism is the same as that in the case of wet-type MCF rubber solar cells.

Unlike the layered structure in a solid-state solar cell consisting of ordered components, the MCF rubber solar cell is a disorderly mixture of NR-latex and MF involving p- and n-type semiconductors and TiO_2_, as a colloidal suspension. Therefore, the conduction of electrons and holes due to photo-excitation is considered to be generated in microscopic sections of the MCF rubber between the isoprene molecule, Fe_3_O_4_ and TiO_2_ particles. Photovoltaic fabrication based on these particles can be considered for numerous synthesized tandem-type solar cells. The photo-production procedure of the solar cell rubber shown here is based on the principle of the ordinary solid-state dye-sensitized solar cell, and the photoreaction process is established in each microscopic section of the MCF rubber. In terms of the electrochemical reaction generated by light, the photo-generated electrons are emitted from the dye pigment, and the oxidized dye is reduced by iodide ion I_3_^−^ with the electrons circulated through the external circuit. The electrons generated from these reactions are transmitted between the microscopic sections one by one, ultimately creating a photocurrent. On the other hand, the magnetic clusters form a bulk hetero-junction structure. 

## 3. Factors of Wet-Type MCF Rubber Solar Cell

**Figure 2:**
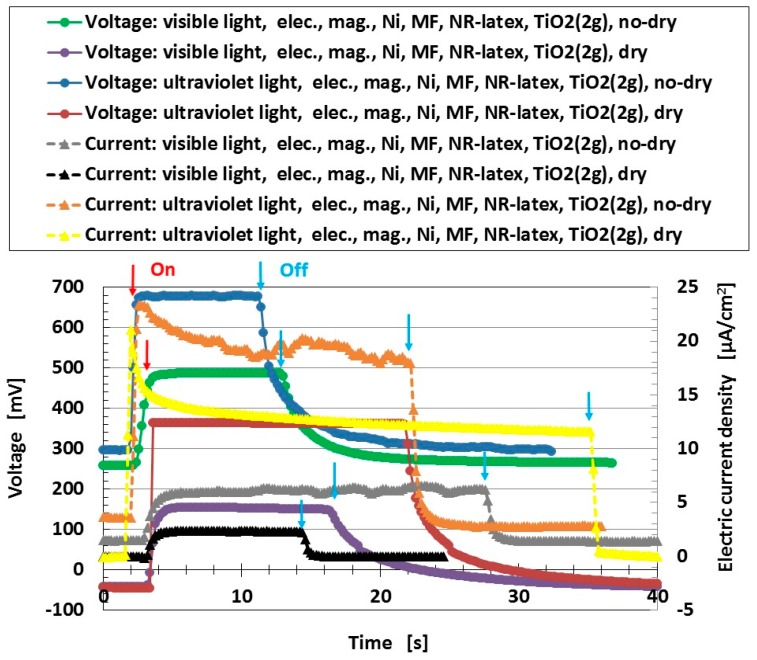
Changes in voltage and electric current density by turning visible and ultraviolet lights on (indicated by red arrow) and off (indicated by blue arrow). “elec.,” electrolytic polymerization; “mag.”, with the magnetic field at electrolytic polymerization; “no-dry,” dye and electrolyte are deposited right before measurement of photoelectricity for wet-type MCF rubber solar cell; “dry,” dye and electrolyte are deposited on MCF rubber after drying it for dry-type MCF rubber solar cell. Each color indicates a component of the MCF rubber solar cell with ruthenium complex dye and electrolyte KI + I_2_.

In this section, we investigate the photoelectricity without tension and compression in order to evaluate the effects of several experimental conditions on a wet-type MCF solar cell rubber. We investigate the difference of photovoltaics between wet- and dry-type MCF solar cell rubbers. Figure 2 shows the changes in voltage and electric current density when visible and ultraviolet light were turned on and off, which are designated by colored arrows in the figure. The MCF solar cell rubber has 2-g TiO_2_, 6-g Ni, 4.5-g MF, 9-g NR-latex, 0.22-g ruthenium complex dye and 7.4-g KI + I_2_. “No-dry” denotes the electrolytically polymerized MCF rubber on which dye and electrolyte are deposited right before measurement under irradiation, so that the rubber is wet. It is so called the “wet-type.” On the other hand, “dry” is the data when the dye and electrolyte are deposited on MCF rubber after drying it under a constant temperature of 105 °C for 1 h in constant temperature drying machine with evaporation of 0.03 g water. As explained in the first report, they possess photoelectric properties including built-in electricity. The former is indicated as a change of the start-up voltage or electric current by lighting and the latter as the initial voltage or electric current before lighting. The built-in voltage refers to the common piezoelectric effect. In addition, built-in current also occurs. The cause is the integrated data at the microscopic part of the adjacent particles or molecules. 

From the figure, the voltage and electric current density of the no-dry type has a larger value than that of the dry type. This result indicates that in the case of arid MCF solar cell rubber, it can be repeatedly used if dye and electrolyte are deposited on it.

**Figure 3:**
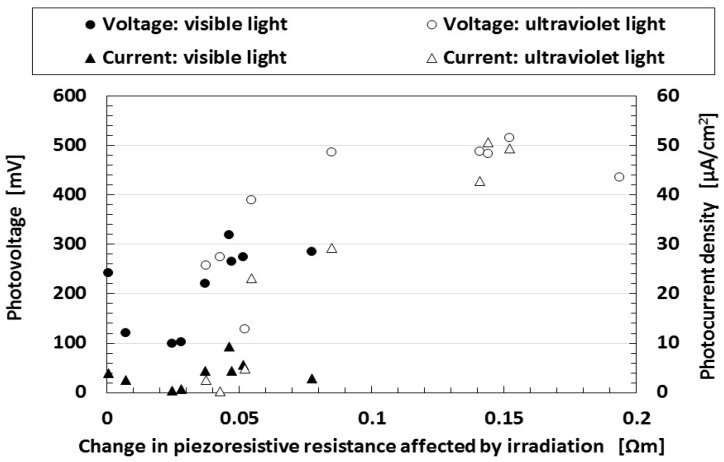
Change in photoelectricity under visible and ultraviolet light on the different kinds of dyes used for wet-type MCF rubber solar cell electrolytically polymerized under a magnetic field. The abscissa in the figure presents the change in the apparent resistance by irradiation.

**Figure 4:**
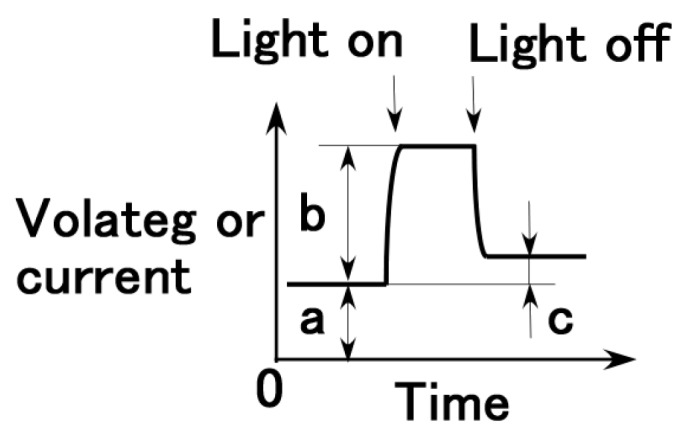
Denoted photoelectricity (photovoltage and photocurrent) as “b”, built-in electricity (built-in voltage and current) as “a”, and charge of built-in voltage and current as “c” which is presented in the consecutive 3rd report on the voltage and electric current curves.

The result that the electric current density obtained in the first report is relatively small is due to the parched surface of the dry-type MCF solar cell rubber. Another potential cause may be the kind of sensitized dye used. Figure 3 shows the effect of dye on photoelectricity when visible and ultraviolet lights were turned on and off, which reflect the changes in the voltage and electric current density by irradiation. Many dyes, Dye 1–10 as shown in Table 1, consist of each combination by the following chemicals: not only ruthenium complex dye but eosine solution (0.44-g eosine Y (C_20_H_8_Br_4_O_5_Na_2_, Fujifilm Wako Pure Chemical Co., Ltd.) and 1.76-g water), mercurochrome solution (merbromin, Kozakai Pharmaceutical Co., Ltd., Tokyo, Japan), iodide lead solution (0.5-g PbI_2_ (lead(II) iodide, Tokyo Chemical Industry Co., Ltd., Tokyo, Japan), 0.5-g methylammonium iodide (CH_5_N·HI, Fujifilm Wako Pure Chemical Co., Ltd.), 7-g water), benzophenone solution (0.32-g benzophenone (C_6_H_5_COC_6_H_5_, Fujifilm Wako Pure Chemical Co., Ltd.), 1.88-g ethyl alcohol), as shown in Table 1. MCF solar cell rubber has 2-g TiO_2_, 6-g Ni, 4.5-g MF, 18-g NR-latex, and 7.4-g KI + I_2_. The abscissa in the figure represents the change in the apparent resistance by irradiation, which means the difference of the apparent resistance between light on and off. This is because the apparent resistance increases due to the chemical reaction of the components of the MCF rubber solar cell under illumination, as previously described in Section 2. In general, the resistance results from the change in the electric current to the application of a voltage and is attributed to the piezoresistivity. It is the so-called apparent resistance which is distinct from the specific resistance of the material. Piezoresistivity is in contrast with piezoelectricity which is due to the change in built-in electricity. Therefore, piezoresistive resistance corresponds to the apparent resistance of the material, and the resistance obtained from the measured electric current at the application of a voltage. Experimental data of voltage and electric current such as in Figure 2 are the direct ones of solar cell generated by irradiation. They are not open and short circuit ones. From these data, photoelectricity (photovoltage and photocurrent) is denoted as “b” (peak voltage and electric current) in Figure 4, built-in electricity (built-in voltage and current) as “a”, and charge of built-in voltage and current as “c” which is presented in the consecutive 3rd report.

**Figure 5:**
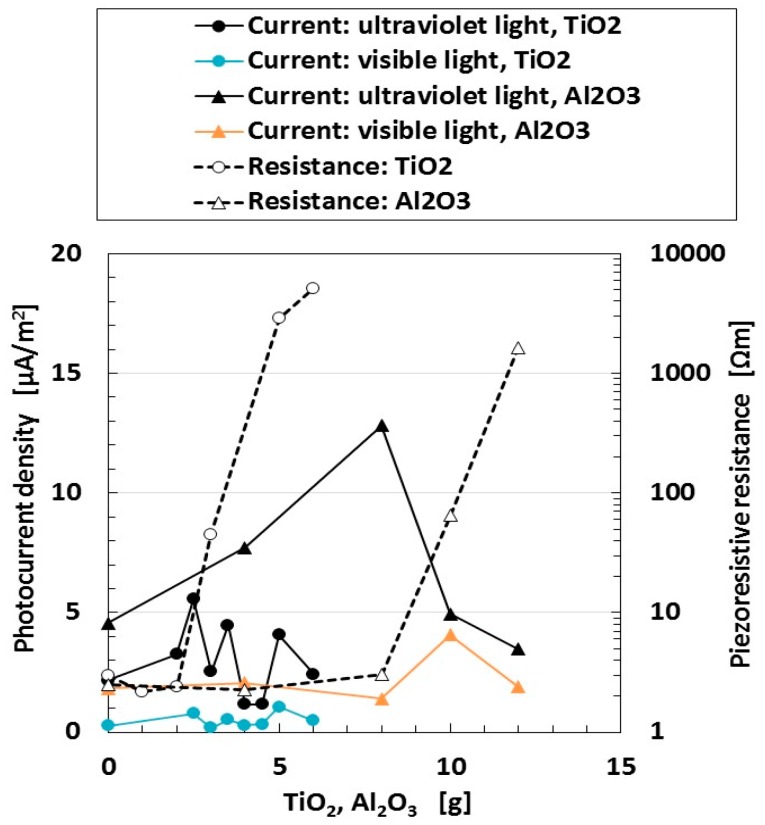
Change in photocurrent density and piezoresistive resistance under visible and ultraviolet light versus the amount of Al_2_O_3_ and TiO_2_ for wet-type MCF rubber solar cell electrolytically polymerized under a magnetic field with ruthenium complex dye and KI + I_2_ electrolyte. The right ordinate in the figure presents the specific resistance at non-irradiation.

Because of the type of dye used, the photocurrent density is larger than that obtained in the first report. Therefore, we can obtain larger photoelectric effects by careful selection of the kind of dye. On the other hand, as the change in the piezoresistive resistance is increased by irradiation, the photoelectricity increases. That is, the larger the change in the apparent resistance by irradiation, the larger the photoelectricity. However, we must note that the change in the apparent resistance in the figure implies a variation in the relative magnitude of the apparent resistance due to irradiation, which is different from specific resistance in the following Figure 5.

We then investigate the absolute quantity of the apparent resistance of MCF rubber solar cells. In order to change its resistance, it is best to incorporate dielectric particles into the MCF rubber, following a previously reported method [29]. Figure 5 shows the photocurrent density and the piezoresistive resistance for wet-type MCF rubber solar cell when visible and ultraviolet light were turned on. 

MCF solar cell rubber has 6-g Ni, 4.5-g MF, 0.22-g ruthenium complex dye and 7.4-g KI + I_2_. In the case of TiO_2_, we used 9-g NR-latex, and for Al_2_O_3_, we used 18-g of NR-latex. The piezoresistive resistance presented in the right ordinate in the figure denotes the specific resistance at non-irradiation. By changing the amount of Al_2_O_3_ and TiO_2_, we can change the specific resistance of the MCF rubber solar cell. From the figure, there is no correlation between photoelectricity and specific resistance. This typical tendency is different from the result of Figure 3. As a result, whether the specific resistance of the MCF rubber solar cell at non-irradiation is small or not, the photocurrent density does not usually become larger according to the amount of specific resistance.

**Figure 6:**
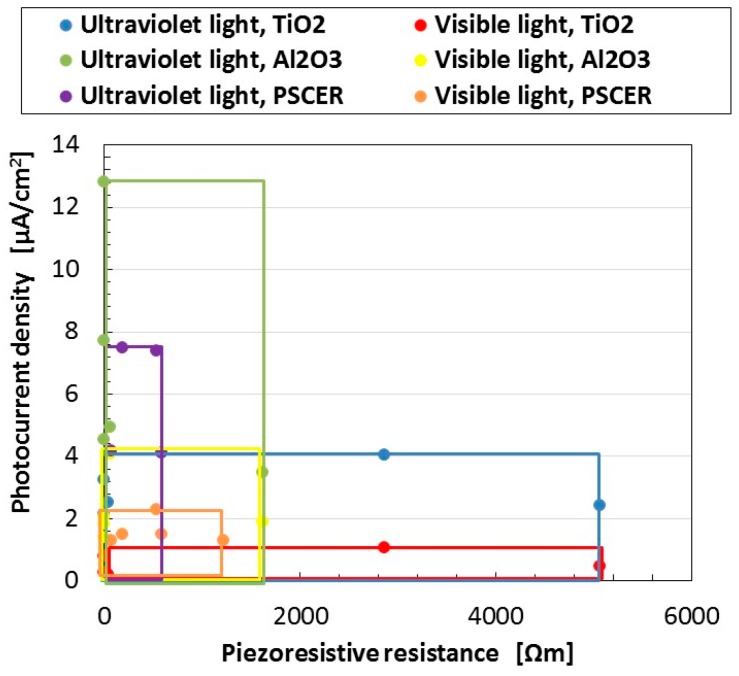
Change in photocurrent density with piezoresistive resistance under visible and ultraviolet irradiation for PSCERs and wet-type MCF rubber solar cells with Al_2_O_3_ and TiO_2_ electrolytically polymerized under a magnetic field with Ruthenium complexes dye and KI + I_2_ electrolyte. Each color indicates the range of values taken by several rubbers at each component. The abscissa in the figure presents specific resistance values at non-irradiation.

From the result of Figure 5, we can raise the question of whether a commercial pressure-sensitive electrically conductive rubber (PSECR) can be used as a wet-type solar cell. Figure 6 shows the photocurrent density to piezoresistive resistance for wet-type MCF rubber solar cells with Al_2_O_3_ and TiO_2_ when visible, and ultraviolet light were turned on, which are the same as those in Figure 5, compared to several PSECRs made of NR-latex produced by a Japanese company (B-1001 (Irumagawa Rubber Co., Ltd., Sayama, Japan), BMSE1-50 (Misumi Group Inc., Tokyo, Japan), AAH65032 (Shimonoseki Packing Co., Ltd., Shimonoseki, Japan), NR Sheet 65 (Fuso Rubber Co., Ltd., Hiroshima, Japan), REP-2 (Tigers Polymer Corp., Osaka, Japan)). On the PSECR as well as the MCF rubber, 0.22-g ruthenium complex dye and 7.4-g KI + I_2_ were poured. The piezoresistive resistance of the abscissa represents the specific resistance for non-irradiation as described in Figure 5. Each colored rectangular indicates the range of values taken by several rubbers for each component. PSCERs are feasible for use in the production of a rubber-type solar cell. The photo-production procedure of PSCERs is based on the principle of the ordinary solid-state dye-sensitized solar cell, and the photoreaction process is established on each surface of the rubber: in terms of the electrochemical reaction generated by light, the photo-generated electrons are emitted from the dye pigment, and the oxidized dye is reduced by iodide ion I_3_^−^ with the electrons circulated through the external circuit, as shown by Equations (12) and (13) in the first report [24]. This corresponds to the conduction of electrons and holes in the case of MCF rubber solar cells due to the photo-excitation generated in microscopic sections of the MCF rubber between the isoprene molecule, Fe_3_O_4_ and TiO_2_ particles. This is because the MCF rubber solar cell is a disorderly mixture of NR-latex and MF involving p- and n-type semiconductors and TiO_2_, such as a colloidal suspension. In the case of PSCER, electrons and holes may be conducted throughout the material. Therefore, we can consider that there may exist the same conductions of electrons and holes in the inner PSCER as those in the case of the MCF rubber solar cell. 

**Figure 7:**
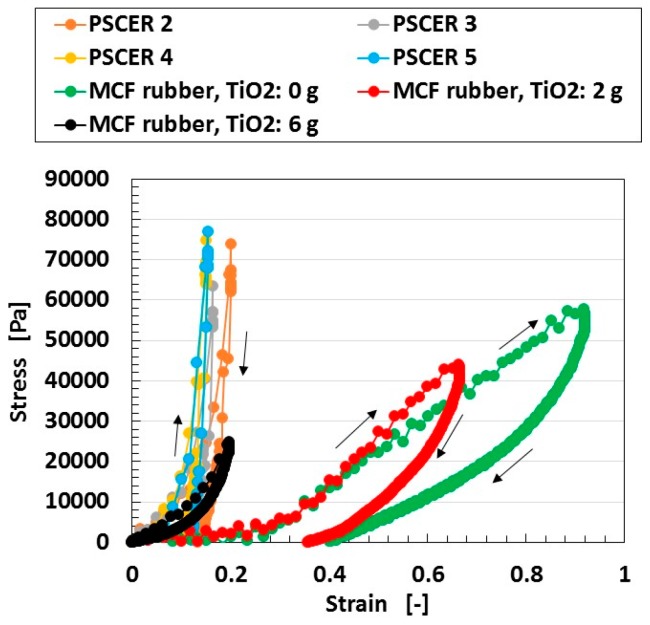
Relation between strain and stress of MCF rubber and PSCERs without dye and electrolyte by tensile testing machine. Wet-type MCF rubber solar cell with TiO_2_ was electrolytically polymerized under a magnetic field. Arrows indicate the progress from tension to its removal.

In Figure 6, the photocurrent density does not necessarily become larger at smaller specific resistance such as that of the PSCERs, which is the same tendency shown in Figure 5. However, PSCER is harder than MCF rubber as shown by the results in Figure 7. 

Nevertheless, the MCF rubber becomes harder as increasing amounts of TiO_2_ are incorporated. MCF rubber is a soft material because of the compounding metal particles such as Ni and Fe_3_O_4_ coated by oleic acid as a filler to the NR-latex, which has stress-strain responses of a well-known hysteresis curve and stress softening (Mullins effect) as shown in a previous study [28,30]. The MCF solar cell rubber has 6-g Ni, 4.5-g MF, 9-g NR-latex, and dye and electrolyte were not used. The response has a five-times-repeated compression and release. The results of the 2-nd stress-strain response of the tensile MCF rubber and the several kinds of PSCERs made of NR-latex are shown in the figure. We used a commercial small-size tensile testing machine (SL-6002, IMADA-SS Co., Ltd., Toyohasi, Japan) as indicated in the previous report [28,30]. All test specimens had a thickness of 1 mm, width of 10 mm, and length of 10 mm at the initial stage before tension was introduced. The maximum tensile force was 0.5 N, and the tensile speed was 100 mm/min. The stress-strain response was non-linear, and a hysteresis curve was obtained because of the filler of Ni and Fe_3_O_4_ coated by oleic acid as shown by the arrows in the figure, which indicate the progress from tension to its removal.

Because PSCER is harder than MCF rubber, PSCER is unable to satisfy the original objective set out in the present study with respect to the development of an artificial soft H-Skin. Furthermore, MCF rubber can generate a larger photocurrent density by using any filler such as Al_2_O_3_.

**Figure 8:**
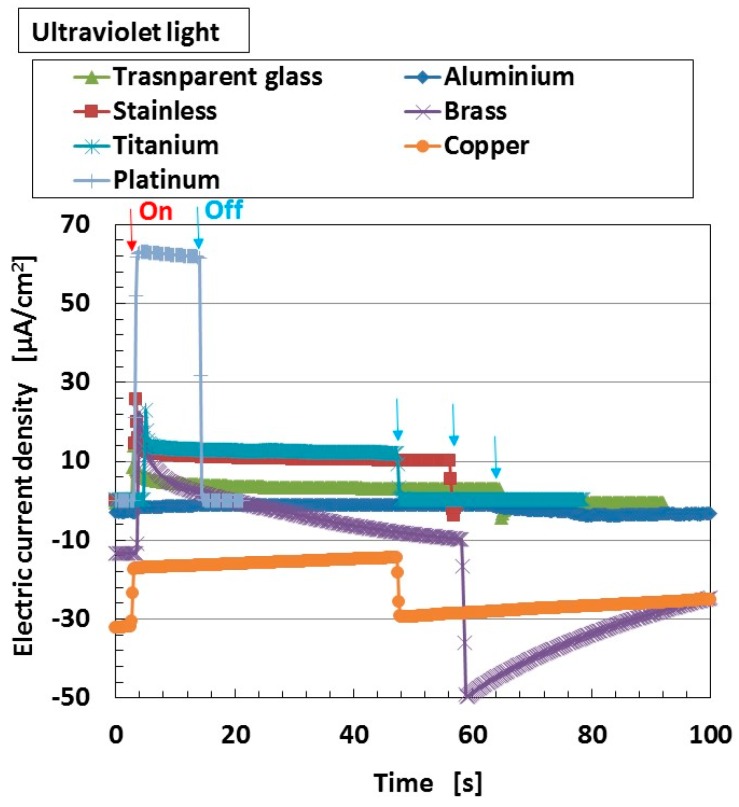
Change in electric current density with ultraviolet irradiation on (indicated by red arrow) and off (indicated by blue arrow) for anode electrode material for wet-type MCF rubber solar cell electrolytically polymerized under a magnetic field with ruthenium complex dye and electrolyte KI + I_2_.

We investigated the effect of the kind of anode electrode material caused in work function on the photovoltaics for wet-type MCF rubber solar cell. Figure 8 shows the changes in the electric current density when ultraviolet light was turned on and off which, and are designated by colored arrows on the anode electrode material in the figure. MCF solar cell rubber has 4-g TiO_2_, 6-g Ni, 4.5-g MF, 9-g NR-latex, 0.22-g ruthenium complex dye and 7.4-g KI + I_2_. We could obtain the same qualitative tendency at irradiation of visible light as that in Figure 8, which is not shown. The tendency that the photovoltaics varies due to the value of the work function is similar to the one for the ordinary Gratzel-type and solid-state solar cells. By selecting optimal material of the anode electrode, we can obtain larger photoelectricity for the wet-type MCF rubber solar cell.

## 4. Tension and Compression Experiments

**Figure 9:**
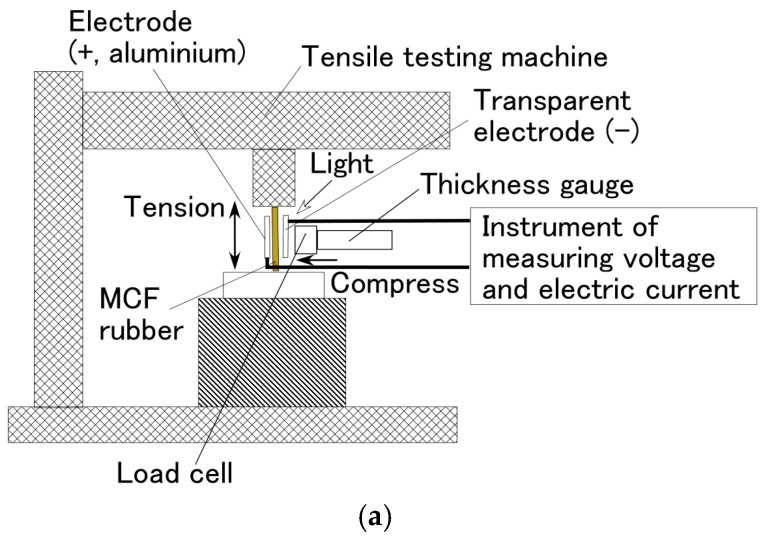

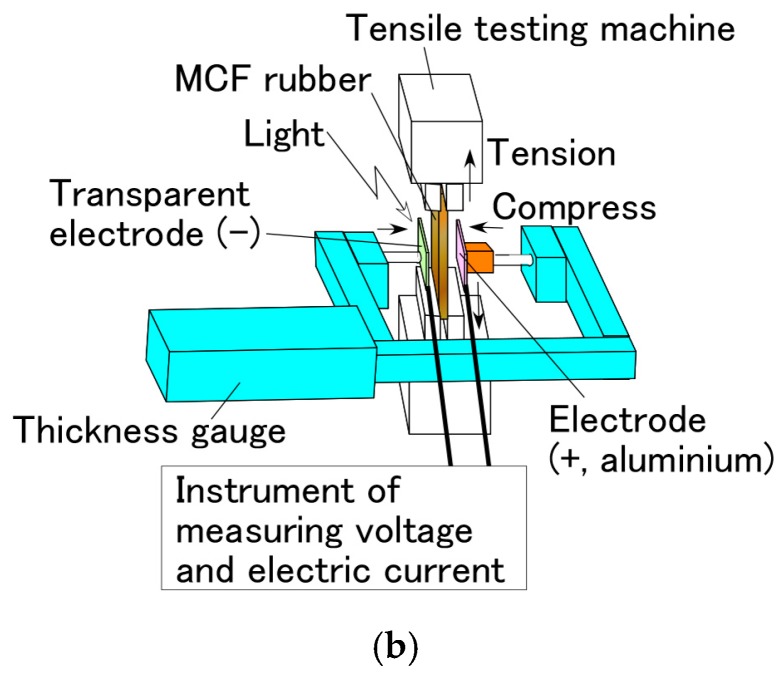
Schematic diagram of experimental apparatus for measuring photoelectricity and piezoelectricity of wet-type MCF rubber solar cell under tension and compression: (**b**) is viewed at an angle on (**a**).

In this section, we investigate the effect of simultaneous excitation of tension and compression on the photovoltaics of the wet-type MCF rubber solar cell. The MCF rubber electrolytically polymerized with 0.22-g ruthenium complex dye on one side and 7.4-g KI + I_2_ on the other was set on a small SL-6002 automatic measuring tensile testing machine (IMADA-SS, Co., Ltd., Toyohashi, Japan) as shown in Figure 9. The production procedure, conditions and the size of the MCF rubber are the same as those described previously in Section 2. A transparent glass (20 mm × 30 mm) coated with TiO_2_ was irradiated with ultraviolet light and connected to the cathode of the solar cell. The MCF rubber was elongated vertically by the tensile testing machine, and at the same time compressed by a thickness gauge transverse to the rubber. For compression, the irradiated glass was compressed by a cylinder with φ 6.5 mm connected to the thickness gauge. Therefore, the irradiated area of the MCF rubber was the area that remained outside of its circular area. The irradiation light was ultraviolet (40 Lux). The voltage and electric current between the electrodes were measured using a PC710 digital multimeter (Sanwa Co., Ltd., Okayama, Japan).

**Figure 10:**
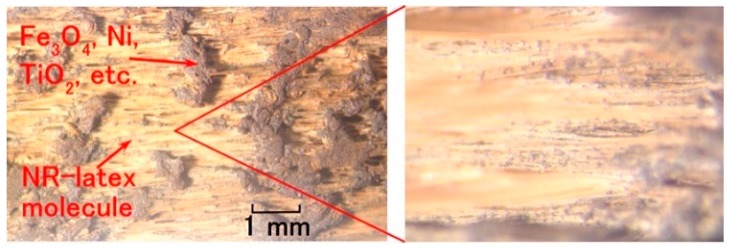
Photographs of surface of elongated MCF rubber electrolytically polymerized with Ni, MF, NR-latex and TiO_2_ before addition of dye and electrolyte at 0.8 of extension strain.

Figure 10 shows the aspect of the MCF rubber under elongation at a maximum tension in the experimental apparatus at 0.8 extension strain. As a result of the elongation, the Fe_3_O_4_, Ni, and TiO_2_ particles were detached from the MCF rubber, and the NR-latex molecule could be confirmed by many fine white lines. However, except at the maximum extension strain of 0.8, detached particles were not observed during the experiment.

**Figure 11:**
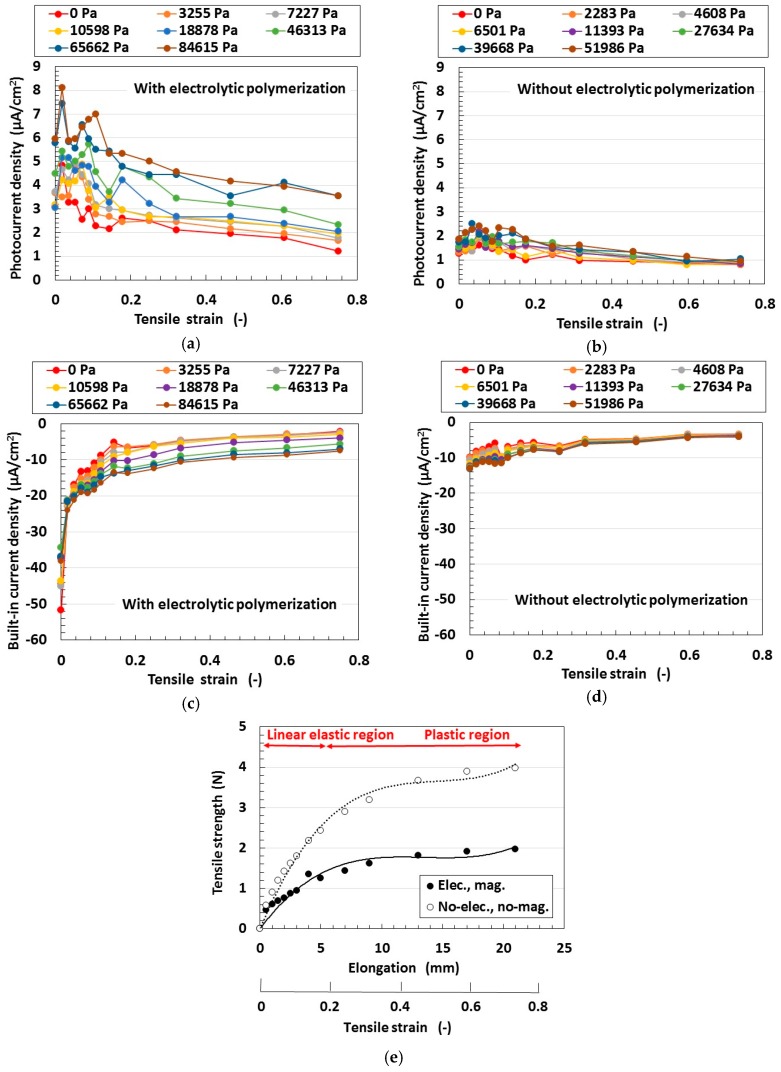
Changes in the electrolytic polymerization effect due to tension under irradiation with ultraviolet light: (**e**) represents the relation between the tensile strength and elongation; elongation rates in various cases (**a**–**d**) for wet-type MCF rubber solar cell electrolytically polymerized under a magnetic field with Ruthenium complexes dye and electrolyte KI + I_2_.

**Figure 12:**
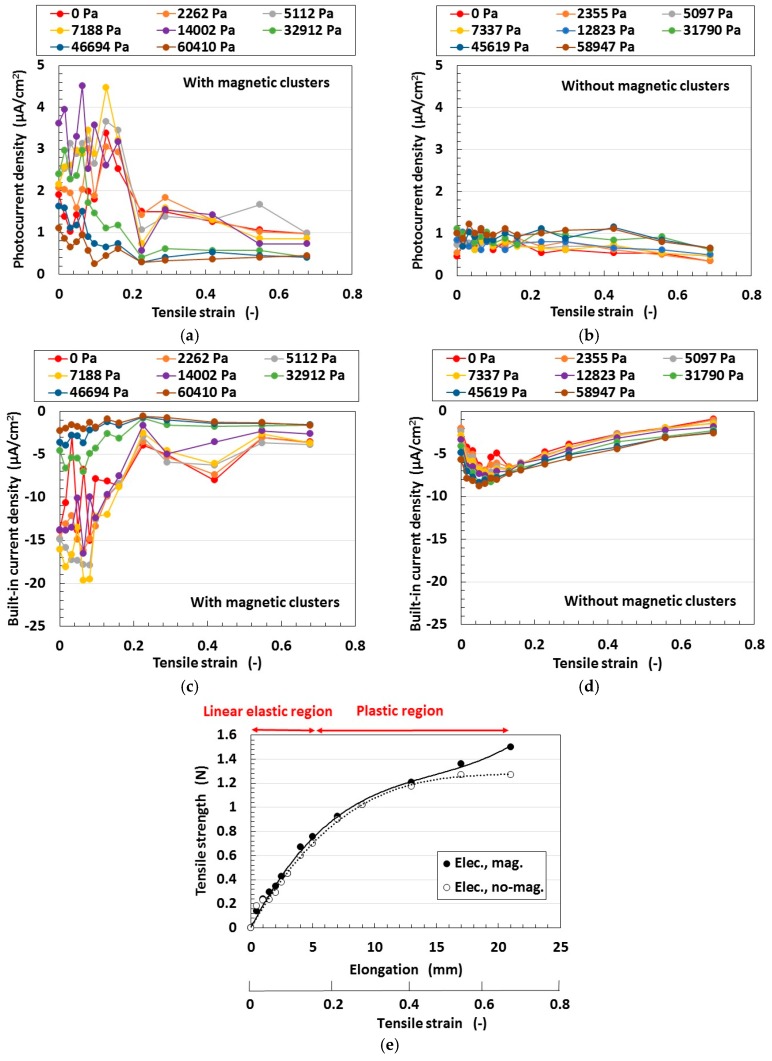
Changes in the effect of magnetic clusters by tension under irradiation with ultraviolet light: (**e**) shows the relation between the tensile strength and the elongation for the various cases from (**a**–**d**) for wet-type MCF rubber solar cell electrolytically polymerized under a magnetic field with Ruthenium complexes dye and electrolyte KI + I_2_.

**Figure 13:**
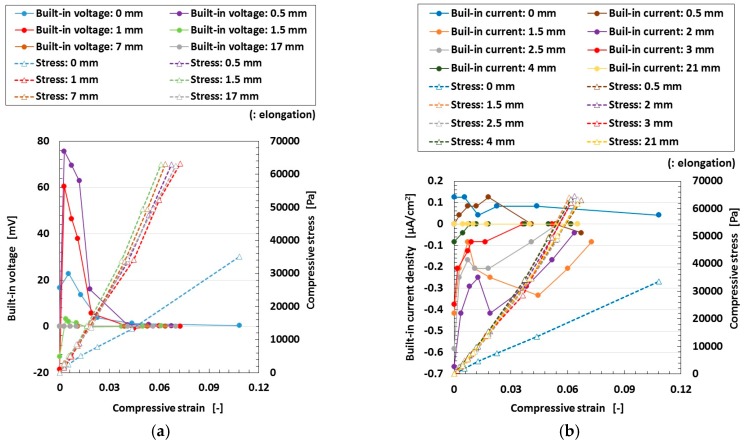

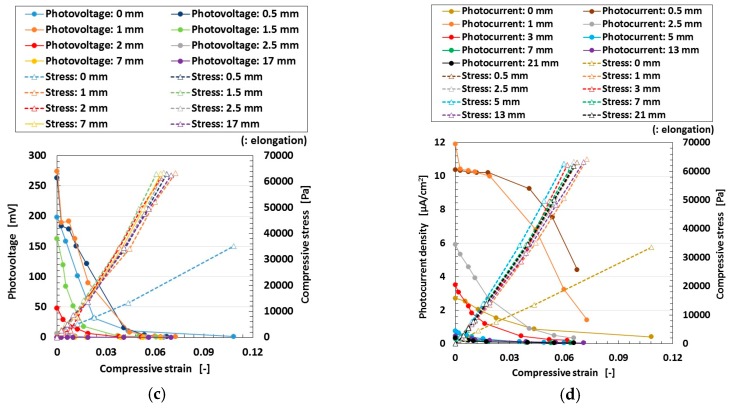
Changes in the effect of compression on the built-in electricity and photoelectricity under ultraviolet irradiation: (**a**,**b**) represent the built-in electricity; (**c**,**d**) represent the photo-electricity; (**a**,**c**) are voltage values; (**b**,**d**) are electric current values for wet-type MCF rubber solar cell electrolytically polymerized under a magnetic field with Ruthenium complexes dye and electrolyte KI + I_2_.

**Figure 14:**
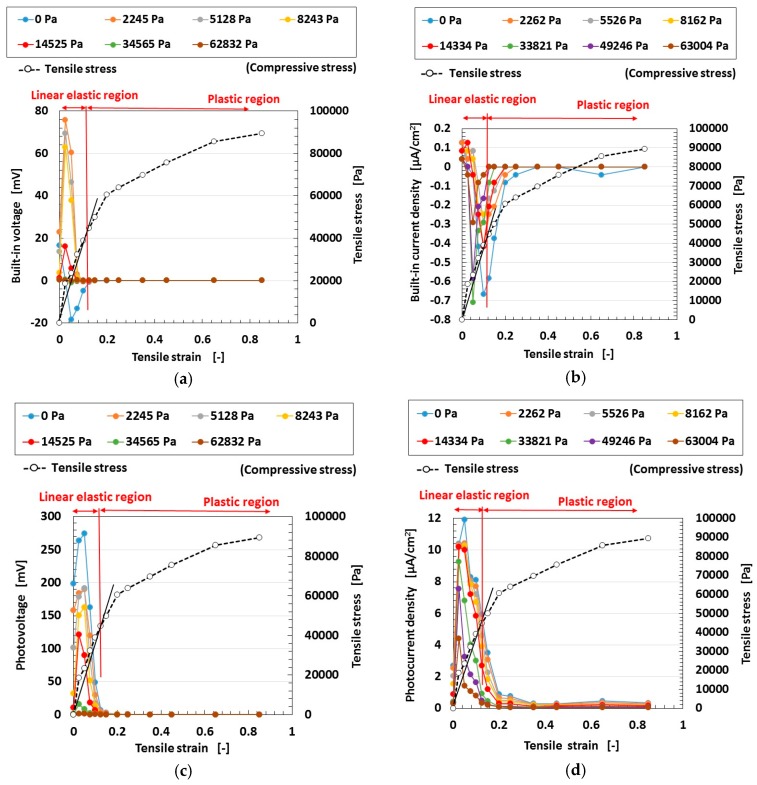
Changes in the effect of the tension on the built-in electricity and photoelectricity under ultraviolet irradiation: (**a**,**b**) represent built-in electricity; (**c**,**d**) represent photo-electricity; (**a**,**c**) are voltage values; (**b**,**d**) are electric current values for wet-type MCF rubber solar cell electrolytically polymerized under a magnetic field with Ruthenium complexes dye and electrolyte KI + I_2_.

At first, we investigated the effect of electrolytic polymerization on the tensioned and compressed MCF rubber solar cell. The change of the electrolytic polymerization effect on the built-in current density as well as the photo current density due to the effect of tension is investigated and the results are shown in Figure 11. Here the MCF rubber had 5-g TiO_2_, 6-g Ni, 4.5-g MF and 9-g NR-latex. The values of pressure indicated above each figure represent compression held constant under elongation, which is also represented in the following cases in Figure 12, Figure 13 and Figure 14. The sole effect of either compression or tension is designated as the case of “0 Pa” of pressure in Figure 11, Figure 12 and Figure 14 for sole effect of tension, or the case of “0 mm” of tension in Figure 13 for sole effect of compression. The built-in electricity is extracted from changes in the voltage and electric current density due to irradiation. The change in the photocurrent density and built-in current density due to elongation increases due to electrolytic polymerization as a result of the aligned formation of the NR-latex molecule and Fe_3_O_4_ particles, etc. On the other hand, increasing the elongation causes the positive photocurrent density to decrease together with the negative built-in current density at a constant pressure. At a constant elongation rate, increasing compression increases the positive photocurrent density and negative built-in current density. These tendencies can be understood by considering the absolute values. They are caused by the approach of the microscopic sections of the MCF rubber. As the distance between the electron and hole, or between the D^+^ and A^−^ ions is narrowed, the electric current is increased.

Secondly, we investigated the effect of magnetic clusters on tensioned and compressed MCF rubber solar cells. By fabrication magnetic clusters, the hetero-junction structure was obtained in the solar cell. Figure 12 compares the effect of the hetero-junction structure on the photoelectricity and built-in electricity. The MCF rubber has 4-g TiO_2_, 6-g Ni, 4.5-g MF and 9-g NR-latex. At first, the change in the photocurrent density and the built-in current density by elongation is larger with magnetic clusters than without. This is due to the fabrication of the hetero-junction structure. At a constant elongation rate, the positive photocurrent density is decreased together with the negative built-in current density by increasing the compression. This tendency can be understood by considering the absolute values. These results are due to the collapse of the hetero-junction structure due to elongation and compression. 

On the other hand, at a range of less than about 0.2 of the elongation rate, the MCF rubber is in the inner linear elastic region. In this region, changes in the photocurrent density and built-in current density by elongation are larger than at the plastic region. This is because the hetero-junction structure can be broken up more easily due to elongation and compression at the plastic state than in the linear elastic region. In particular, at the linear elastic region the photocurrent density and built-in current density have peak values; i.e., they are increased once. These are the typical characteristics of the MCF rubber solar cell. We can utilize this tendency in engineering applications to obtain higher efficiency solar cells by elongating the MCF rubber in the inner linear elastic region. Incidentally, this characteristic can also be observed in Figure 11 and Figure 12. 

Thirdly, we investigated the effect of tension and compression on the MCF rubber solar cell. The change of the built-in electricity and photoelectricity by compression and tension are shown in Figure 13 and Figure 14, respectively. Here the MCF rubber had 3-g TiO_2_, 6-g Ni, 4.5-g MF and 9-g NR-latex.The built-in electricity and photoelectricity decreased for larger compression. The maximum compressive strain range is smaller than that in the case of tension because the thickness of the compressed rubber is small, such as 1-mm order. Therefore, the deformation of the compression is in the inner linear elastic region.

The built-in electricity and photoelectricity increase once and then decreased as the tension was increased. There exist peaks in the inner linear elastic region. In the plastic region, the built-in electricity and photoelectricity are minimal. It is a significant engineering application regarding H-Skin installed in a robot, and elastic and flexible solar cell to utilize the typical peaks in the inner linear elastic region because we can obtain larger photoelectricity by tension. 

Regarding tension, the MCF rubber has linear elastic and plastic regions as shown in Figure 11, Figure 12 and Figure 14 during our used tensile condition. It does not have damage by mechanical force inner linear elastic region, and its configuration holds without breaking. Therefore, the same properties including photovoltaics are repeatable. However, at plastic region, it is transformed and then the many properties including photovoltaics are changed. In contrast, as for compression, the reformation is inner linear elastic region because the reformation by the compression is confined inner thin thickness of the MCF rubber as shown in Figure 13. This phenomena can be seen in ordinary rubber material. And then the configuration holds without breaking. Therefore, the same properties including photovoltaics are repeatable.

**Figure 15:**
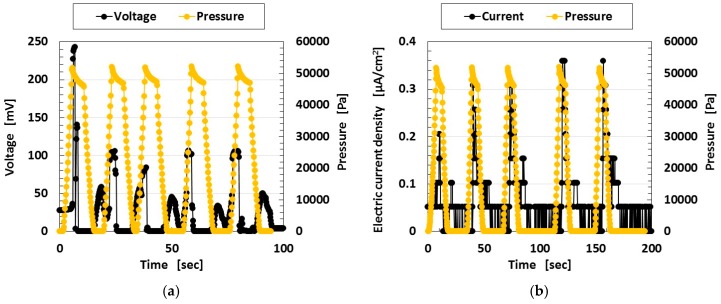
Changes in the voltage and electric current density with pressure repeated five times under ultraviolet irradiation: (**a**) voltage; (**b**) electric current density for wet-type MCF rubber solar cell electrolytically polymerized under a magnetic field.

**Figure A1:**
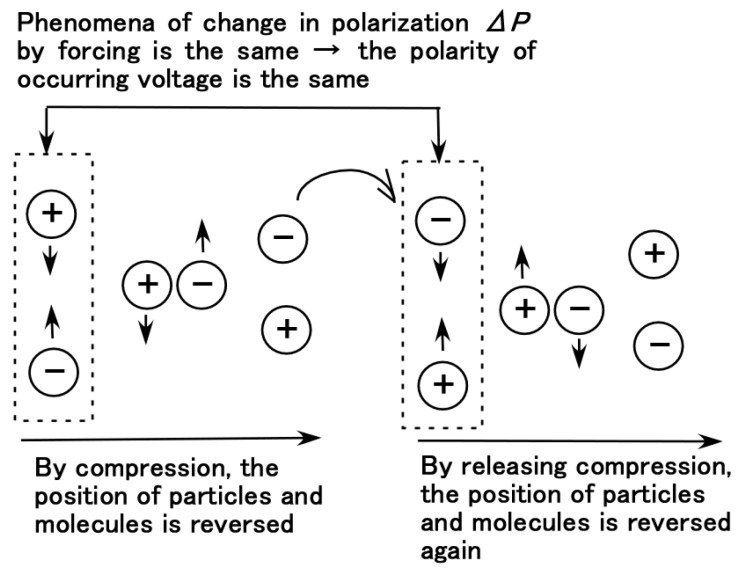
Schematic diagram of change in configuration of ionized particles and molecules at the part of MCF rubber by compression and release.

In the case of compression, the MCF rubber solar cell corresponds to a piezo element. Figure 15 shows the changes in the voltage and electric current when pressure is applied to the MCF rubber solar cell, which implies built-in electricity. The electrolytically polymerized MCF rubber was sandwiched between a transparent glass electrode and a TiO_2_-coated one (20 mm × 30 mm). Ultraviolet light was scattered on the transparent electrode coated with TiO_2_. A force of up to 10 N was applied by the rod with a diameter of φ 8 mm, which was in contact with the electrode on one side under the incident light, and then removed. The experimental apparatus is the same as in the first report. The rod is attached to a commercial small-size tensile testing machine SL-6002 which is used as shown in Figure 7. The compression speed was 10 mm/min, and the compression movement was repeated five times. The voltage and electric current between the electrodes were measured. When the pressure is small in the range of the initial period of compression and the last period of removal, the built-in voltage increases with compression. However, when the pressure is larger than the value at these ranges, the built-in electricity decreases. This tendency is the same as that of the piezo sensor made of MCF rubber as shown in the previous study [26] and in Figure A1 in Appendix A. According to the previous study [26], the effective piezoelectric coefficient d^33^ is estimated to be on the order of 10^−10^ C/N which was measured by referring the metrics in other work, and as for piezoresistivity, the electric conductivity ranged from 10^−4^ to 10^−1^ S/m. In addition, they were compared with other piezo-materials. If we use the MCF rubber, the solar cell is able to behave as a piezo element.

## 5. Effect of Electrode Position

Next, we considered the effect of different positions with a fix distance between the electrodes of the solar cell on the photovoltaics. In this section, we deal with the case without tension and compression for the wet-type MCF rubber solar cell. 

**Figure 16:**
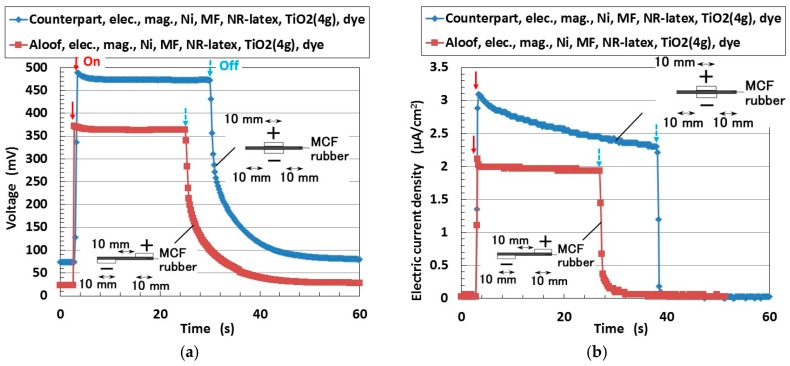
Changes in voltage and electric current density under ultraviolet light on (indicated by red arrow) and off (indicated by blue arrow) by electrode location for wet-type MCF rubber solar cell electrolytically polymerized under a magnetic field with Ruthenium complexes dye and electrolyte KI + I_2_: (**a**) voltage; (**b**) electric current density.

At first, we address the condition of short distance between the electrodes of the solar cell. Figure 16 shows the changes in the voltage and electric current density when ultraviolet light (227 Lux) was turned on and off. The MCF rubber was electrolytically polymerized with 0.22-g ruthenium complex dye on one side and 7.4-g KI + I_2_ on the other. The MCF rubber had 4-g TiO_2_, 6-g Ni, 4.5-g MF and 9-g NR-latex. The production procedure, conditions and the size of the MCF rubber are the same as those described previously in Section 2. In the figure, “Counterpart” and “Aloof” refer to the different locations of the solar cell electrodes. The MCF rubber had an area of 16 mm × 30 mm and was 1-mm thick. Both electrodes were 20 mm × 10 mm. The irradiation light was ultraviolet (40 Lux). In this investigation, the voltage and electric current between the electrodes were measured.

In the case of ordinary solid-type solar cells, the location of the electrodes is a counterpart to that of the material. Therefore, the photovoltaic reaction is along the thickness of the material. In contrast, to the MCF rubber solar cell, even if the electrodes are apart, the photovoltaic reaction occurs. Electrodes can thus be set anywhere. This is due to the mixture of the components of the MCF rubber and the conduction of electrons and holes in microscopic sections of the rubber among the isoprene molecule, Fe_3_O_4_, and TiO_2_ particles. Therefore, photo-excitation can be generated wherever the electrodes are set on the MCF rubber. This tendency was only recently reported in ordinary solar cells. The feasibility of this concept in MCF rubber solar cells depends on the solid-like rubber material produced by electrolytic polymerization. It is suitable for engineering applications of new types of solar cells, for example, the crooked and deformed solar cell with installed atwain electrodes.

**Figure 17:**
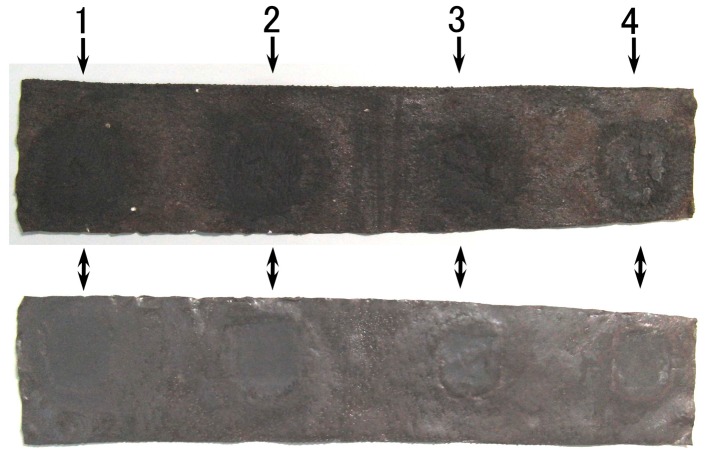
Photographs of obverse and reverse surfaces of wet-type MCF rubber solar cell electrolytically polymerized under a magnetic field. The upper photograph is the cathode side at electrolytic polymerization (UC), and the lower surface is the anode side (LA).

Next, we deal with the condition of long distance between electrodes of a solar cell with multiple electrodes as shown in Figure 17. Four 188-mT neodymium permanent magnets, which are the same as those in Figure 1, were applied to the 50 mm × 240 mm MCF rubber under electrolytic polymerization at each of the positions 1–4. The MCF rubber had 2-g TiO_2_, 6-g Ni, 4.5-g MF and 9-g NR-latex. The dark-colored mark shown in the figure are at the location of the applied magnets. Those may be the location of electrodes of solar cell. The production procedure, conditions and the size of the MCF rubber are the same as those described the previously in Section 2. 

**Figure 18:**
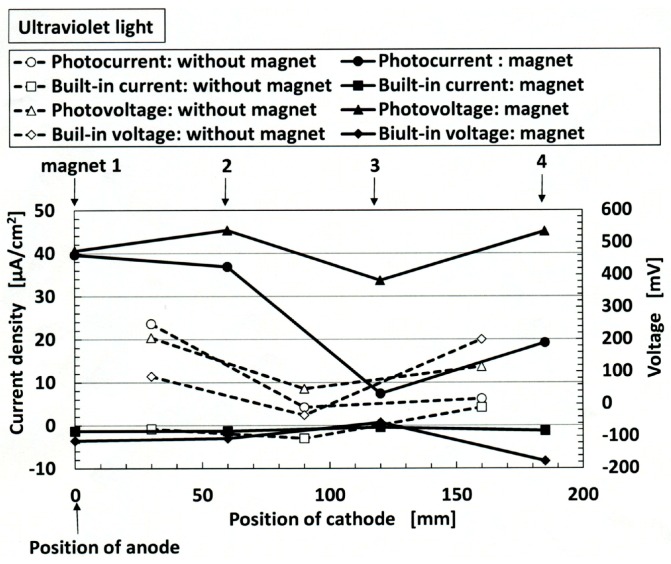
Change in photoelectricity and built-in electricity under ultraviolet irradiation on the position of the cathode of the solar cell for wet-type MCF rubber solar cell electrolytically polymerized under a magnetic field with ruthenium complex dye and electrolyte KI + I_2_: “without magnet” indicates the position between the regions influenced by the permanent magnet at electrolytic polymerization; “with magnet” represents the position influenced by the permanent magnet at electrolytic polymerization. In the figure, positions 1–4 for the applied magnet at electrolytic polymerization are also shown.

A transparent glass (20 mm × 30 mm) and a second one coated with TiO_2_ were placed in contact with UC and LA respectively, as shown in Figure 17. KI + I_2_ was deposited On UC 7.4-g, and ruthenium complex dye was deposited on LA 0.22-g. UC is connected as the anode of the solar cell, and LA as the cathode. Ultraviolet light was irradiated on LA (227 Lux). Figure 18 shows the photoelectricity and built-in electricity. The anode of the solar cell is fixed at position 1 while the cathode of the solar cell is changed from position 1 to position 4 as shown in the figure. Even if the distance between the electrodes of the solar cell increases, photoelectricity can be generated. The photoelectricity at the position of “magnet” is larger than at the position of “without magnet.” This is due to the magnetic clusters aligned at the position of application of the magnet. On the other hand, built-in electricity at the position of “magnet” as well as at the position of “without magnet” is generated, however, small by comparison to photoelectricity.

**Figure 19:**
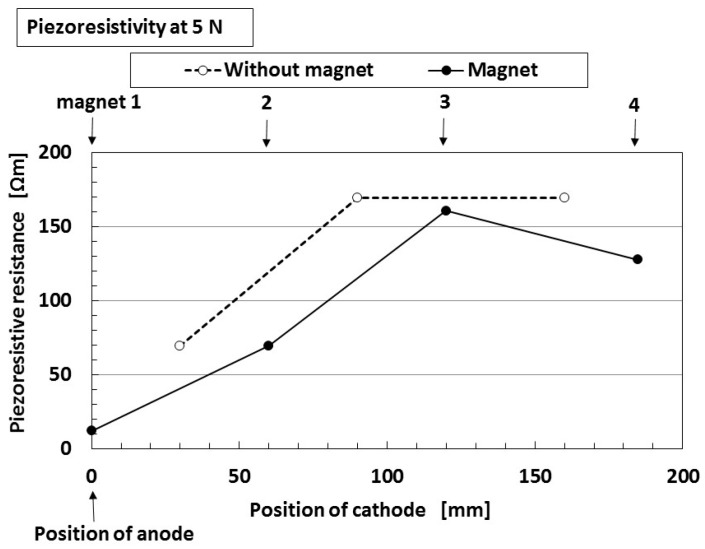
Change in the piezoresistance by the position of the cathode for wet-type MCF rubber solar cell electrolytically polymerized under a magnetic field: “without magnet” indicates the position between the regions that are influenced by the permanent magnet at electrolytic polymerization; “with magnet” indicates the position influenced by the permanent magnet at electrolytic polymerization. In the figure, positions 1–4 of the applied magnet at electrolytic polymerization are also shown. The ordinate in the figure presents specific resistance values at non-irradiation.

We considered the effect of the increase in the resistance with an increase of the separation distance of the electrodes, on the photoelectricity and built-in electricity. Figure 19 shows the piezoresistive resistance between the anode and cathode of the solar cell. It denotes specific resistance values at non-irradiation, which is the same as that in Figure 5. The anode of the solar cell is fixed at position 1, and the cathode of the solar cell is changed from position 1 to position 4 as shown in the figure. Naturally, as the distance between the electrodes increases, the piezoresistive resistance becomes larger. The piezoresistive resistance at the position of “magnet” is smaller than at the position of “without magnet.” This is due to the magnetic clusters aligned at the position of application of a magnet.

According to the results from Figure 18 and Figure 19, the following conclusions can be obtained: the ordinate of Figure 19 denotes inherent resistance of the MCF rubber, and shows that the resistance becomes larger as the distance between cathode and anode becomes longer. On the other hand, the built-in voltage and built-in current shown in Figure 18 denote piezoelectricity, and the figure shows that we can obtain the piezoelectricity even if the distance between cathode and anode becomes longer. By combining these results, we can obtain the photoelectricity, even if the MCF rubber solar cell and the distance between the electrodes are large so that the specific resistance also becomes large.

## 6. Conclusions

In order to realize a solar cell with flexible, elastic and extensible properties, we fabricated a solar cell using the conventional principles of an electrolyte system in wet-type or dye-sensitized solar cells as a consecutive study of the first report. The photovoltaic effect was accomplished with electrochemical photolysis by the combination of water and TiO_2_, referring to the Honda-Fujishima effect, and by utilizing electrolytically polymerized MCF rubber, with a magnetic field associated with the creation of magnetic clusters. MCF rubber solar cell is defined by the synthesis of a dye-sensitized solar cell and an organic thin-film solar cell as well as a tandem-type solar cell. The wet-type MCF solar cell rubber was produced via the synthesis of TiO_2_ particles, and MCF rubber with NR-latex and MCF through compounding of MF and Ni particles, dye, and electrolyte. The photovoltaic- and piezo- effects were generated as the total transmission of electrons and built-in voltage in each of the microscopic sections, which served as a mechanism based on the principle of ordinary solid-state dye-sensitized solar cell and the photoreaction process. The wet-type MCF rubber solar cell created both photovoltaics and piezoelectricity. In the present report, the effects of electrodes distance as well as the simultaneous existence of tension and compression on the photovoltaics were evaluated. We classify the main accomplishments of this study as follows:

As for Section 3:

- It was determined that the photoelectricity of wet-type MCF rubber solar cell is larger than that of the dry-type. Even if the MCF rubber is dry due to the electrolytic polymerization, photovoltaic- and piezo-electricity can be generated as long as dye and electrolyte are deposited next to the MCF rubber. MCF rubber solar cell can be used repeatedly by depositing electrolyte and dye. Therefore, it can be considered to have both states of dry and wet rubber and is effective for long-term use. The creation of a flexible, elastic, and extensible solar cell could generate many new engineering applications in the fields of robotics, etc. Therefore, MCF rubber solar cells can be expected to improve the daily lives of individuals. Incidentally, there is yet room for improvement in the kind of electrolyte and liquid dye in the quest to develop higher generated power.
- Increasing change in resistance by irradiation, results in an increase in photoelectricity. However, there is no correlation between photoelectricity and specific resistance as piezoresistive resistance.
- PSCERs are also feasible for use as a rubber-type solar cell. However, PSCER is harder than MCF rubber.

As for Section 4:

- Changes to the photocurrent density and built-in current density by elongation were increased by electrolytic polymerization. By increasing elongation under constant pressure, the photocurrent density and built-in current density decreased; however, increasing the compression under constant elongation rate led to an increase of their values.
- For elongated MCF rubber solar cells, the photocurrent density and built-in current density were larger with magnetic clusters than without. By increasing compression under a constant elongation rate, the photocurrent density and the built-in current density decreased.
- Changes in photocurrent density and built-in current density by elongation were larger than in the plastic region. In the linear elastic region of the MCF rubber, the photocurrent density and built-in current density exhibited a peak value and are increased once.

As for Section 5:

- In contrast to the ordinary solid-state solar cell, the photovoltaic- and piezo-electricity of the MCF rubber solar cell can be generated even if the electrodes are apart. Electrodes can be set anywhere in the MCF rubber solar cell. Even if MCF rubber solar cell and the distance between the electrodes are large so that the specific resistance becomes larger, we can still generate photoelectricity and piezo-electricity.

Finally, the interesting problem of how much the MCF rubber solar cell can charge electricity by irradiation remains. Because the existence of built-in voltage between A^−^ and D^+^ can be assumed to be related to the electric charge. In addition, in order to investigate the characteristics of the MCF rubber solar cell, the measurement with cyclic voltammograms is another effective means, because the wet-type MCF rubber solar cell uses a sensitized dye and electrolyte such as Gratzel-type solar cell and its photovoltaics depends on oxidation-reduction reactions. This will be dealt with in the next 3rd report. The electric current-voltage curves of the MCF rubber solar cell is also significant information for the solar cell field, however, it will be described in other report. 

## Figures and Tables

**Table 1:** Components of used 10 types dye by combination of several solutions for wet-type MCF rubber solar cell.

	Dye 1	Dye 2	Dye 3	Dye 4	Dye 5	Dye 6	Dye 7	Dye 8	Dye 9	Dye 10
Ruthenium complexes dye	0.22 g									2.4 g
Eosine solution		2.2 g				2.2 g		2.2 g	2.2 g	
Mercurochrome solution			3.65 g			7.39 g	7.39 g		7.39 g	2.4 g
Iodide lead solution				8 g						5.32 g
Benzophenone solution					2.2 g		2.2 g	2.2 g	2.2 g	

## Appendix A

As for Figure 15, we can confirm the typical characteristics of the same polarity both at compression and at release, which differs from ordinary ferroelectric or piezoelectric materials. The phonomena already been clarified experimentally in the previous study [26]. MCF shows piezoelectric-like behavior without involving piezoelectric material. Where MCF involves ferroelectric material, which is magnetic particles Fe_3_O_4_ coated by oleic acid. When the opposing ionized particles and molecules approach each other by compression, polarization *P* decreases by change in polarization *ΔP* according to their smaller slippage *Δδ*, and then occurring voltage changes by compression. In addition, there can be other case that their positions are reversed because of larger compression, as shown in the following figure. The cause is due to the elastic configuration of natural rubber with the dispersion of particles and molecules. The configuration of the particles and molecules in solidified natural rubber is different from the one of ordinary ferroelectric or piezoelectric material. In the case of their reverse positions, the change in *ΔP* according to *Δδ* by releasing compression is the same as the one by compression. In addition, as for MCF rubber, the polarity must be considered to be the sum of the change in *ΔP* owing to their positions of ionized particles and molecules. Therefore, the polarity at releasing compression is the same as the one at compression. Thus, the same tendency of opposite polarity of occurring voltage as in the ordinary ferroelectric or piezoelectric materials does not always occur.
